# *Haemophilus parasuis*: infection, immunity and enrofloxacin

**DOI:** 10.1186/s13567-015-0263-3

**Published:** 2015-10-28

**Authors:** Nubia Macedo, Albert Rovira, Montserrat Torremorell

**Affiliations:** College of Veterinary Medicine, University of Minnesota, 1988 Fitch Ave, St. Paul, MN 55108 USA

## Abstract

*Haemophilus parasuis* is an early colonizer of the porcine upper respiratory tract and is the etiological agent of Glasser’s disease. The factors responsible for *H. parasuis* colonization and systemic infection are not yet well understood, while prevention and control of Glasser’s disease continues to be challenging. Recent studies on innate immunity to *H. parasuis* have demonstrated that porcine alveolar macrophages (PAMs) are able to differentially up-regulate several genes related to inflammation and phagocytosis, and several pro-inflammatory cytokines are produced by porcine cells upon exposure to *H. parasuis*. The susceptibility of *H. parasuis* strains to phagocytosis by PAMs and the bactericidal effect of complement are influenced by the virulent phenotype of the strains. While non-virulent strains are susceptible to phagocytosis and complement, virulent strains are resistant to both. However, in the presence of specific antibodies against *H. parasuis*, virulent strains become susceptible to phagocytosis. More information is still needed, though, in order to better understand the host immune responses to *H. parasuis*. Antimicrobials are commonly used in the swine industry to help treat and control Glasser’s disease. Some of the common antimicrobials have been shown to reduce colonization by *H. parasuis*, which may have implications for disease dynamics, development of effective immune responses and immunomodulation. Here, we provide the current state of research on innate and adaptive immune responses to *H. parasuis* and discuss the potential effect of enrofloxacin on the development of a protective immune response against *H. parasuis* infection.

## Introduction

*Haemophilus parasuis* is one of the most important bacteria affecting pigs. The disease caused by this pathogen is characterized by polyserositis and it is known as Glasser’s disease [1]. *H. parasuis* is present in all major swine-rearing countries and remains a significant pathogen in contemporary swine production systems [1]. In addition to causing disease, *H. parasuis* is frequently isolated from the upper respiratory tract of healthy pigs [2, 3]. Multiple different genotypes and serotypes of *H. parasuis* have been described. However, there is not a clear association between virulence and *H. parasuis* phenotypes or genotypes [4]. Successful vaccination resulting in decreased mortality has been achieved by bacterins and autogenous vaccines, but failures are frequent due to poor cross-protection [5–8]. The ability of *H. parasuis* to interact with the swine host, causing or not disease, is a subject that needs further investigation. Recently, reverse vaccinology and immunoproteomic analysis identified several putative virulence-associated genes and immunogenic proteins in different *H. parasuis* strains [9–12]. Follow-up vaccine studies in mice and piglets using recombinant antigens revealed strong seroconversion, but only partial protection against homologous challenge and weak or inexistent cross-protection [13, 14].

Because of the incomplete efficacy of vaccines, antimicrobials are needed to treat *H. parasuis* infections [1]. Pigs receiving antimicrobials early during infection with *H. parasuis* are usually able to survive a systemic infection [1]. More specifically, enrofloxacin is a fluoroquinolone active against Gram-negative and Gram-positive bacteria [15]. Enrofloxacin inhibits the bacterial DNA gyrase (a type II topoisomerase), preventing DNA supercoiling and replication, which leads to cell death [16]. Additionally, enrofloxacin has been shown to temporarily decrease the load of *H. parasuis* naturally colonizing the upper respiratory tract of conventional pigs [3].

Even though there is not a standard method for evaluating the antimicrobial susceptibility against *H. parasuis* [17], some studies that included Spanish [18] and Chinese [19] strains have shown antimicrobial resistance to enrofloxacin using breakpoints recommended by the Clinical and Laboratory Standard Institute (CLSI) for other bacterial species. Although many *H. parasuis* strains are considered susceptible to enrofloxacin, it is important to emphasize the judicious use of antimicrobials to treat Glasser’s disease and to monitor susceptibility patterns of *H. parasuis* isolates before administration of a given therapy.

Enrofloxacin has also been shown to hinder immunity to several bacterial species, including *Actinobacillus pleuropneumoniae* in swine [20]. Moreover, early elimination of various bacterial pathogens by antimicrobials hindered the development of protective immune responses necessary to overcome future infections [21–23]. While it is clear that the use of antimicrobials exert a direct deleterious effect over bacterial infections, recent findings described below are shedding light on their potential effect on immune responses. However, the interaction between antimicrobials and immune responses to *H. parasuis* is not known. The purpose of the present review is to summarize existing knowledge concerning the swine immune response to *H. parasuis* and we discuss the potential mechanisms for interaction between enrofloxacin and immunity.

## Protective immunity against *H. parasuis*

There has been a great expansion on knowledge in regards to the pig immune system and its effect in disease and protective immunity against infections. Pigs can respond almost immediately to an infectious agent through innate immune mechanisms, which might control the infection until activation of the adaptive immune system [23]. Shortly after infection, bacteria encounters the innate immune system, which is activated when pattern-recognition receptors (PRRs), including toll-like receptors (TLRs), contact pathogen-associated molecular patterns (PAMPs) and induce different signaling pathways [24]. Bacterial invasion also triggers the complement system [25], induces migration of phagocytic cells and the production of various cytokines, which provides antimicrobial defense, recruit of more cells through the inflammatory process and assists in the activation of acquired immunity [23].

The activation of the acquired immune response results in additional cytokine production, T-cell and B-cell activation, and antibody production. The acquired immune response also provides the host with specific memory for protection against subsequent homologous infections [23]. Specific reagents, improved technology and more detailed knowledge of the porcine immune cell populations now enable detailed analyses of the antigen-specific immune responses in swine [26–29]. Although protective immunity against extracellular bacteria is largely dependent on antibodies, cellular responses are often required for full expression of immunity [23].

### Innate defense mechanisms to *H. parasuis*

Porcine alveolar macrophages (PAMs) are considered an important line of defense against *H. parasuis* infection [30]. PAMs isolated from pigs inoculated with *H. parasuis* were able to differentially up-regulate several genes related to cytokine production, phagocytosis, formation of phagolysosome, signal transduction and nitric oxide production [31].

In vitro studies have demonstrated that non-virulent strains are susceptible to phagocytosis by PAMs, while virulent *H. parasuis* strains are resistant [30]. Differently from the mechanism of phagocytosis for non-virulent strains, phagocytosis of virulent strains is not dependent on actin filaments [30]. In addition, competition assays have shown that phagocytosis of *H. parasuis* is probably not dependent on a specific receptor, since phagocytosis of non-virulent strains was not affected by the presence of non-virulent or virulent strains [30].

Moreover, in vivo studies have shown that there is a delay in the processing of virulent *H. parasuis* strains by PAMs and a 24 h delay in macrophage activation by *H. parasuis* virulent strains when compared to non-virulent strains [32]. While there is no difference on association of virulent and non-virulent strains with early endosomes, non-virulent strains were found more frequently associated with mature endosomes than virulent strains after one-hour incubation [33]. This inhibition of early host responses to virulent *H. parasuis* may lead to the development of Glasser’s disease [32]. Interference of phagocytosis by *H. parasuis* virulent strains is likely associated with presence of capsule [30]. Additionally, two virulent-associated trimeric autotransporter (VtaA) antigens, VtaA 8 and VtaA 9, identified in the *H. parasuis* outer membrane, delayed interactions with macrophages, even though they did not prevent phagocytosis [33].

Immunohistochemistry and immunoperoxidase techniques have also demonstrated that following systemic infection, *H. parasuis* antigens were found as degenerated bacteria in dilated phagosomes in serosal lesions [34, 35]. Apparently, late leukocyte responses that are activated after infection with virulent strains are able to efficiently phagocytize *H. parasuis*, which is in agreement with in vitro studies that have shown that virulent *H. parasuis* strains do not survive inside macrophages when internalized [30].

Cytokines participating in the inflammatory response to *H. parasuis* including interleukin (IL)-8 and IL-6, were produced by porcine tracheal and endothelial cells upon contact with *H. parasuis* [36, 37]. Acute phase response stimulated by IL-6 production and chemoattraction of leukocytes stimulated by IL-8 represent essential roles of these cytokines in inflammatory response to *H. parasuis* [36]. Furthermore, increased IL-1α expression in lung has been reported in pigs undergoing severe Glasser’s disease following experimental infection, whereas IL-4, IL-10, tumor necrosis factor (TNF)-α, and interferon (IFN)-γ were expressed in significantly higher levels in spleen, pharyngeal lymph nodes, lung and brain of survivors [38]. Similarly, in vivo studies with pigs challenged with highly virulent *H. parasuis* showed an increase proportion of CD163+ monocytes, which are able to produce high amounts of proinflammatory cytokines, such as TNF-α, IL-1 and IL-6 [39].

As part of another line of defense of the innate immune response, γδ T-cells were found in reduced numbers in pigs after challenge with a lethal dose of a highly virulent *H. parasuis* strain [39]. γδ T-cells represent a numerous lymphocyte subset population in pigs able to recognize unprocessed non-peptide antigens [26]. These cells can cause cytotoxicity and produce T helper (Th)-1 and Th-2 cytokines that contribute to inflammatory and anti-inflammatory immune response [31]. A reduction on γδ T-cells might render the pigs more susceptible to *H. parasuis* infection, suggesting that this could be one of the mechanisms of pathogenesis of *H. parasuis* virulent strains even though such mechanism still needs to be elucidated [39].

Antibody-independent activation of the complement cascade is an initial host’s defense mechanism, causing vasodilatation and increased vascular permeability resulting in attraction of phagocytic cells to the site of infection [23]. The complement cascade also results in the formation of a complex of proteins that acts as a pore in the bacterial wall ultimately causing bacterial lysis. The activation of complement can be made by bacterial endotoxins such as lipopolysaccharides (LPS), peptidoglycan and teichoic acids [23]. Non-virulent *H. parasuis* strains were susceptible to complement in an antibody-independent way, while virulent strains evade this host response and resist to complement-mediated killing [40]. Therefore, resistance to antibody-independent complement killing seems to be a mechanism of pathogenicity of *H. parasuis* virulent strains [40] and it was demonstrated that the *H. parasuis* outer membrane protein P2 (OmpP2) is required for serum resistance [41].

### Acquired defense mechanisms against *H. parasuis*

While the porcine innate immune system confers initial protection, the acquired immune system provides a second, more specific and long lasting, line of defense against infectious organisms [25]. According to studies performed in mice, following antigen stimulation, Th cells differentiate into Th-1 or Th-2 cells. The functions of Th-1 and Th-2 cells correlate with the production of their cytokines. Th-1 cells are involved in cell-mediated inflammatory functions through secretion of IL-2 and IFN-γ. Th-2 cells encourage antibody production, and also enhance eosinophil proliferation and function by secreting IL-4, IL-5, IL-6, IL-9, IL-10 and IL-13 [42]. Accordingly, Th-2 cytokines are commonly found in association with antibody responses [42], even though in pigs the Th-2 cytokine, IL-4, was not able to stimulate porcine B-cell and antibody production in vitro [43].

Significant rises in the total proportion of Th-1 and Th-2 (CD4+) cells were observed in all pigs that survived challenge with live *H. parasuis*, while susceptible pigs showed a decrease of CD4+ T cells [44]. Cytotoxic (CD8+) T cells and B-cells were significantly increased in all pigs between 1 and 3 days after challenge, independently of survival [39]. Further studies are needed to elucidate cellular immune responses to *H. parasuis* infection that are related to protection.

A humoral immune response is usually activated when pigs are infected with *H. parasuis* [35]. Such response is frequently associated with the development of a transient IgM response followed by a solid and progressively increasing IgG antibody response [45]. Moreover, passive immunization of pigs with serum containing specific antibodies against *H. parasuis* confirmed the role of the humoral response on protection against lethal challenge [46]. The mechanism of protection by antibodies seems to be due to the role of antibodies in opsonization of *H. parasuis* strains to facilitate phagocytosis [30]. In particular, virulent *H. parasuis* strains require prior opsonization with specific antibodies in order to be phagocytosed by PAMs and, if internalized, they are successfully killed by PAMs [30].

In comparison with the amount of information available on the immune response to *H. parasuis* systemic infection, knowledge on immune response to *H. parasuis* colonization is limited, even though *H. parasuis* is a common colonizer of pigs. It has been demonstrated that non-virulent *H. parasuis* strains possess mechanisms of immune evasion, such as biofilm formation [47]. Biofilm formation might protect bacteria from the attack of the host immune response and facilitate colonization of the upper respiratory tract by non-virulent strains. In addition, non-virulent strains are usually susceptible to phagocytosis by PAMs [30] and sensitive to the bactericidal effects of serum [48], which might prevent bacteria from surviving in the lungs and invading systemically the hosts. In addition, an increase in *H. parasuis* colonization rate was associated with a decrease in *H. parasuis* serum antibodies [2]. Therefore, serum antibodies in piglets might be able to modulate the timing and level of colonization by *H. parasuis*, and be relevant to avoid systemic disease caused by *H. parasuis* [2].

## Effects of enrofloxacin on the immune response

Most of the findings reporting the interactions of antimicrobials with the immune system were discovered using mouse models [21, 22]. These findings contribute to the understanding of how the use of antimicrobial drugs can interfere with protection against a specific bacterial infection, either by interacting with the cellular and/or humoral immune response or by decreasing the antigen responsible for triggering an immune response. However, the mechanisms by which they act still need to be further investigated, especially for large animals.

Enrofloxacin, a fluoroquinolone, is actively accumulated in phagocytes, but it did not interfere with the chemotaxic action of porcine polymorphonuclear leukocytes (PMNs) or phagocytosis of *A. pleuropneumoniae*, *Pasteurella multocida* and *Staphylococcus aureus* by PMNs and PAMs when compared to untreated controls [49]. However, the intraphagocytic killing of *A. pleuropneumoniae* was significantly enhanced by enrofloxacin in both PMNs and PAMs [49]. More research is needed, though, to investigate whether these effects would also apply to *H. parasuis*.

In swine, a protective humoral immune response to *A. pleuropneumoniae* was impeded by treatment with enrofloxacin but not by treatment with penicillin or tetracycline [20]. Absence of seroconversion might have been related to the high efficiency of enrofloxacin in eliminating the inoculated *A. pleuropneumoniae* quickly enough to prevent the activation of an acquired immune response. Regarding *H. parasuis*, even though there is no specific information available on antimicrobial interference on the immune response, it is known that enrofloxacin is able to reduce the load of *H. parasuis* in the upper respiratory tract of naturally colonized pigs [3]. Interference with *H. parasuis* colonization might be associated with interference with the development of a protective immune response to colonizing bacteria, since pigs experimentally inoculated with a low dose of virulent *H. parasuis* strain were less susceptible to Glasser’s disease in the field [50, 51].

## Conclusions

Protection against *H. parasuis* disease involves the activation of several elements of the innate and acquired porcine immune system, most of which are still unknown. Specific *H. parasuis* virulent factors allow this bacterium to evade the innate immune system and invade systemic tissues, causing severe inflammation of serosas by cytokine activation and attraction of phagocytes. A serum antibody response is usually present in pigs surviving systemic infection or after vaccination and is highly associated with protection against *H. parasuis* disease, even though heterologous protection is limited. Since antimicrobial use is widespread in the swine industry and antimicrobials are used as an option to control *H. parasuis* disease, their effects on the immune response need to be taken into account. While antibiotic treatment can be very effective at controlling *H. parasuis* infections, it may also interfere with the development of protective immune responses against *H. parasuis*. Therefore, an improved understanding of how *H. parasuis* primes a protective immune response and the specific roles of humoral and cellular immune responses in protection to *H. parasuis* disease are needed. An improved understanding on the effect of antimicrobials on the immune response will contribute to the development of better control programs for *H. parasuis* and will help develop judicious antibiotic treatment practices.

## Abbreviations

PRR: pattern-recognition receptor
TLR: toll-like receptor
PAMP: pathogen-associated molecular pattern
PAM: porcine alveolar macrophage
vtaA: virulent-associated trimeric autotransporter
IL: interleukin
TNF: tumor necrosis factor
IFN: interferon
Th: T helper cells
LPS: lipopolysaccharides
Ig: immunoglobulin
PMN: polymorphonuclear leukocytes
